# Comparative virology of HTLV-1 and HTLV-2

**DOI:** 10.1186/s12977-019-0483-0

**Published:** 2019-08-07

**Authors:** Michael P. Martinez, Jacob Al-Saleem, Patrick L. Green

**Affiliations:** 1 Center for Retrovirus Research, The Ohio State University, Columbus, OH USA; 2 Department of Veterinary Biosciences, The Ohio State University, Columbus, OH USA; 3 Comprehensive Cancer Center, The Ohio State University, Columbus, OH USA

**Keywords:** HTLV-1, HTLV-2, Tax, HBZ, APH-2, Adult T-cell leukemia, ATL, HTLV-1 associated myelopathy/tropical spastic paraparesis, HAM/TSP

## Abstract

Human T cell leukemia virus type 1 (HTLV-1) was the first discovered human retrovirus and the etiologic agent of adult T-cell leukemia and HTLV-1-associated myelopathy/tropical spastic paraparesis. Shortly after the discovery of HTLV-1, human T-cell leukemia virus type 2 (HTLV-2) was isolated from a patient with hairy cell leukemia. Despite possession of similar structural features to HTLV-1, HTLV-2 has not been definitively associated with lymphoproliferative disease. Since their discovery, studies have been performed with the goal of highlighting the differences between HTLV-1 and HTLV-2. A better understanding of these differences will shed light on the specific pathogenic mechanisms of HTLV-1 and lead to novel therapeutic targets. This review will compare and contrast the two oldest human retroviruses with regards to epidemiology, genomic structure, gene products, and pathobiology.

## Introduction

In 1980, Poiesz et al. reported the discovery of the first human retrovirus isolated from a patient with cutaneous T-cell lymphoma. This virus is now known as human T-cell leukemia virus type-1 (HTLV-1) [1]. In the years since, several HTLV subtypes have been discovered: HTLV-2 was first identified in a patient with hairy cell leukemia [2], while HTLV-3 and HTLV-4 were discovered in bushmeat hunters in Africa [3, 4]. HTLV is a zoonotic virus with simian T-cell leukemia virus counterparts found in monkeys. HTLV-1 and HTLV-2 are the most well studied subtypes of HTLV. They share roughly 70% nucleotide similarity and have a similar genome structure. Both viruses encode the structural and enzymatic proteins shared by all retroviruses, both encode the regulatory proteins Tax and Rex, and both feature a RNA transcript and protein derived from the negative-sense strand of the viral genome. HTLV-1 and HTLV-2 also express several accessory proteins that support various aspects of virus biology.

HTLV-1 is associated with several diseases, including adult T-cell leukemia (ATL) and HTLV-1 associated myelopathy/tropical spastic paraparesis (HAM/TSP) [5–7]. Interestingly, while HTLV-2 was initially discovered in a patient with hairy cell leukemia, no clinical correlation between HTLV-2 and lymphoproliferative disease has been established [2]. Instances of HTLV-2 infected individuals reporting HAM/TSP-like symptoms have been described, but a clear correlation between the virus and symptoms has not been clinically established [8, 9].

Many studies over the years have dissected differences between HTLV-1 and HTLV-2. Recent years have focused on differences between the regulatory protein Tax and the antisense-derived proteins, HBZ (HTLV-1) and APH-2 (HTLV-2). The different cellular pathways and signaling cascades that these proteins activate likely play a key role in the divergent pathogenic outcomes of these viruses. Studies of HTLV-1 are of high importance due to the diseases associated with the virus. An increase in studies of HTLV-2 would be beneficial to learn what this virus lacks causing it to not be associated with disease. This review will compare and contrast the two oldest human retroviruses and emphasize the differences that exist between these viruses and the potential they may have for treatment of HTLV-1-associated diseases.

## Epidemiology and transmission

There are an estimated five to ten million individuals infected with HTLV-1 worldwide with endemic regions of infection in Southwest Japan, sub-Saharan Africa, South America, the Caribbean, and regions of the Middle East and Australo-Melanesia [10]. The estimate of infected individuals is based on data collected from 1.5 billion individuals within known HTLV-1 endemic regions. Reasonably accurate estimations within some highly populous, traditionally non-HTLV-1 endemic regions have not been completed. Furthermore, large population-based studies of HTLV-1 prevalence are rare and most current studies analyze specific subsets of the population (commonly blood donors or pregnant women). Given these limitations, the number of HTLV-1 infected individuals is likely much higher than the current estimate.

Seroprevalence of HTLV-1 in areas of endemicity is estimated at 1–2%, and was found to reach as high as 20–40% in individuals greater than 50 years of age [10]. A major socio-economically and culturally independent epidemiological determinant of HTLV-1 seroprevalence is age. A 2018 hospital-based cohort study of HTLV-1 infection in an indigenous Australian population documented a progressive increase in seropositive rates with age reaching 48.5% in men 50–64 years of age [11]. Other important determinants of HTLV-1 seroprevalence in endemic regions include gender and economic status [12]. A study examining over 250,000 individuals in an HTLV-1 endemic region found that females had an overall higher seroprevalence than males, with males carrying a higher seroprevalence between the ages of 16–19 years of age and females carrying a comparable or higher seroprevalence over the age of 20 years [13]. Additionally, a more recent retrospective analysis of more than 3 million repeat blood donors in Japan found a much higher incidence of seroconversion in women than in men with an estimated 4190 (3215 women and 975 men) new HTLV-1 infections per year [14]. The association of lower socio-economic status with higher seroprevalence was specifically documented in Jamaica, where the unemployed, farmers, and laborers were found to carry a higher seroprevalence than those reporting student or professional occupations [15].

HTLV-1 demonstrates robust genetic stability. Mapping of stable nucleotide substitutions specific to varied geographic regions has been used to classify virus strains into geographic subtypes [16, 17]. The major geographic subtypes are Cosmopolitan subtype A, Central African subtype B, Australo-Melanesian subtype C, and Central African/Pygmies subtype D. Cosmopolitan subtype A is the most widespread subtype (endemic subgroups in Japan, Central and South America, the Caribbean, North and West Africa, and regions of the Middle East). Central African subtypes E, F, and G exist, but are rare [18].

With an estimated 800,000 infected individuals worldwide, HTLV-2 is far less prevalent than HTLV-1. Most documented HTLV-2 infected individuals are found in the United States (400,000–500,000) highly concentrated in the Native American and intravenous drug user populations. A similar epidemiologic pattern is found in the second most HTLV-2 infected region, Brazil (200,000–250,000). The lower prevalence of HTLV-2 when compared to HTLV-1 reflects specific concentration of infection within Native American groups and intravenous drug users [19]. HTLV-2 is divided into four molecular subtypes; a, b, c, and d. HTLV-2a and HTLV-2b are commonly found in the Americas and Europe whereas HTLV-2c and HTLV-2d are found predominantly in Brazil and Central Africa [20–22].

HTLV-1 and HTLV-2 require cell-to-cell contact for efficient transmission [23]. Both viruses utilize Envelope (Env) glycoprotein-mediated cell binding and entry. The HTLV-1 and HTLV-2 surface (SU) and transmembrane (TM) subunits of Env share 65% and 79% residue identity, respectively [24]. Despite this high similarity, HTLV-1 and HTLV-2 utilize a slightly different complex of receptor molecules. HTLV-1 utilizes heparan sulfate proteoglycan (HSPG) and neuropilin-1 (NRP1) for binding and glucose transporter 1 (GLUT1) for entry. HTLV-2 also utilizes NRP1 and GLUT1, but not HSPGs [25–27].

There are three primary modes of HTLV transmission: vertical (e.g. during parturition or breast feeding), parenteral (e.g. transfusion of contaminated blood products, transplantation of infected organs, or intravenous drug use), and sexual [28–32]. Breastfeeding is the most common route of vertical transmission with risk factors including high breast milk proviral load, high HTLV-1 serum antibody titers, and breast feeding for a duration greater than 6 months [33–36]. HTLV-1 infection via transfusion was first demonstrated by Okochi et al. in Japan [29]. Studies since have found seroconversion rates after transfusion with HTLV-1 positive cellular blood products to range from 12 to 74% under varying conditions [37–39]. HTLV-2 can be transmitted vertically through breastfeeding and horizontally via sexual contact, but is most commonly transmitted via sharing of contaminated needles amongst intravenous drug users [40, 41]. Many countries remain without established screening protocols and prevention campaigns for HTLV.

## Genome structure and gene expression

**Fig. 1:**
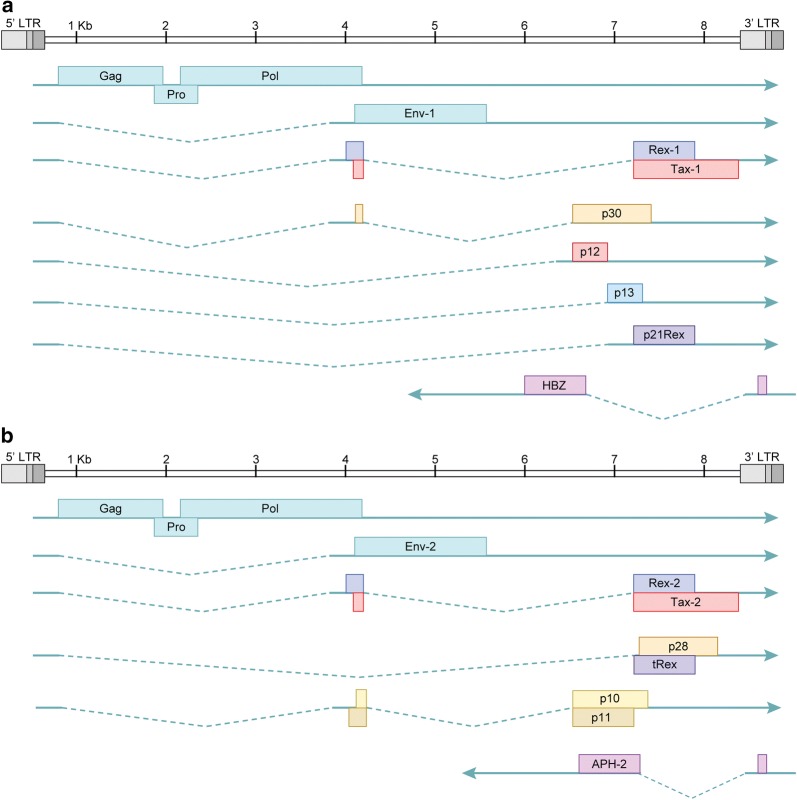
HTLV-1 and HTLV-2 genomes and transcripts. **a** HTLV-1 genome, transcripts, and associated proteins. **b** HTLV-2 genome, transcripts, and associated proteins

HTLV is a member of the delta retrovirus family. These viruses are complex retroviruses that express regulatory and accessory genes, in addition to the structural and enzymatic genes common to all retroviruses. The proviral genomes of HTLV-1 and HTLV-2 are depicted in Fig. 1a, b. Both genomes are roughly 9 kb in length and feature 5′ and 3′ long terminal repeats (LTR), which are direct repeats generated during the reverse transcription process. The 5′ portions of both genomes encode the structural and enzymatic gene products (Gag, Pol, Pro, and Env). The regulatory and accessory genes are expressed from the historically termed ‘pX’ region of the genome. The pX region is located 3′ of the structural gene *Env*. Both HTLVs encode an antisense gene, *HBZ* for HTLV-1 and *APH*-*2* for HTLV-2, located on the negative or minus strand of the proviral genome.

After integration of the proviral genome, several different HTLV transcripts will be produced (Fig. 1a, b show a summary of these transcripts). Both viruses utilize the viral regulatory protein Tax and the viral promoter located in the 5′ LTR to drive viral gene transcription. The viral protein Rex ensures export of the unspliced viral mRNAs. The unspliced full-length viral mRNA serves as the viral genome for future virions and also as the source of Gag, Pol, and Pro proteins. Several different splice variant mRNAs are also expressed to generate Env, the regulatory proteins, and the accessory proteins. Expression of the antisense genes of HTLV is not regulated by the Tax or Rex proteins, but is instead dependent on host cellular factors to promote transcription. The next few sections will cover the different proteins expressed by HTLV-1 and HTLV-2.

## Tax-1 and Tax-2

**Fig. 2:**
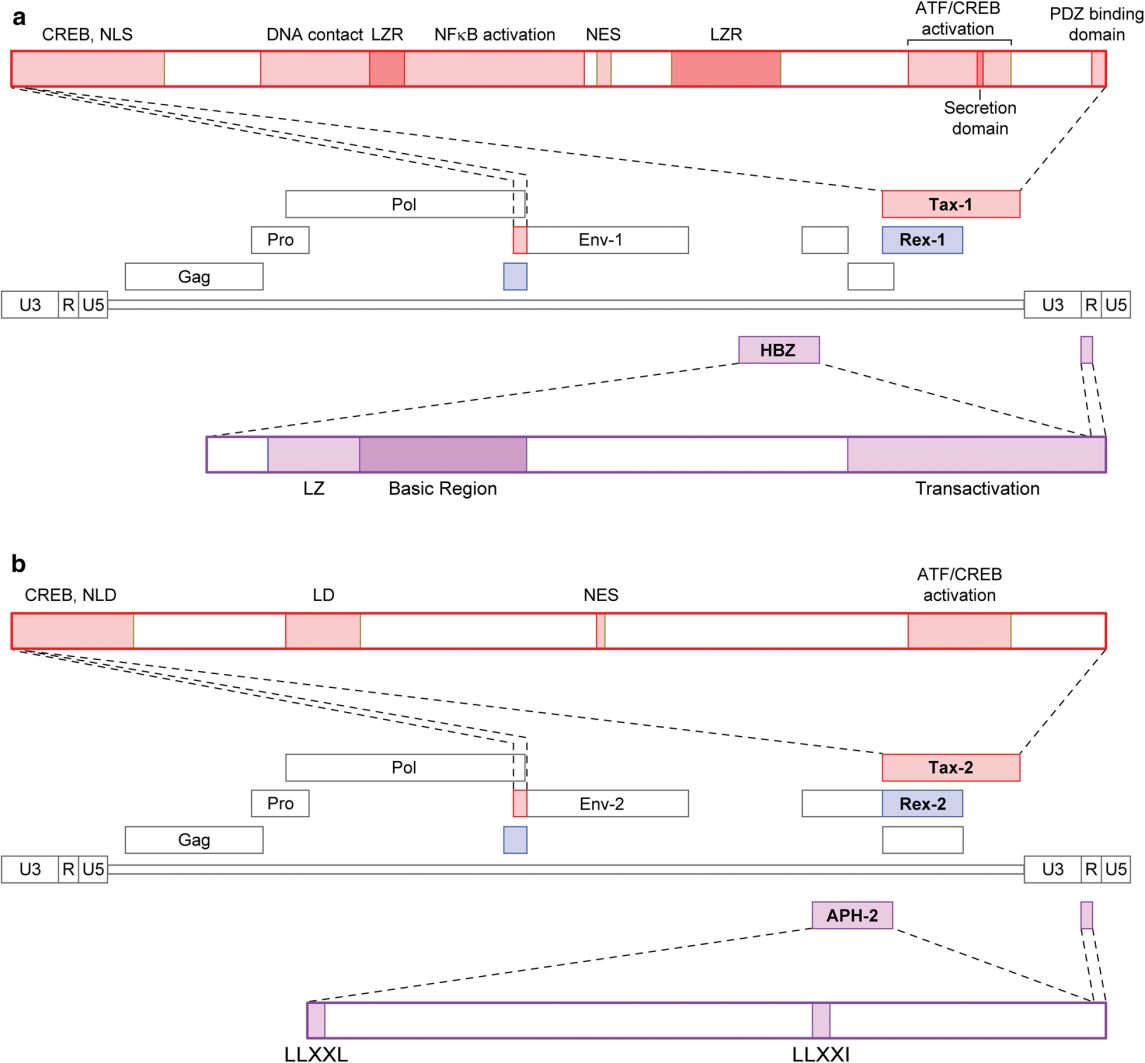
Functional domains of Tax-1, Tax-2, HBZ, and APH-2. **a** HTLV-1 protein products and functional domains Tax-1 and HBZ. **b** HTLV-2 protein products and functional domains of Tax-2 and APH-2

**Fig. 3:**
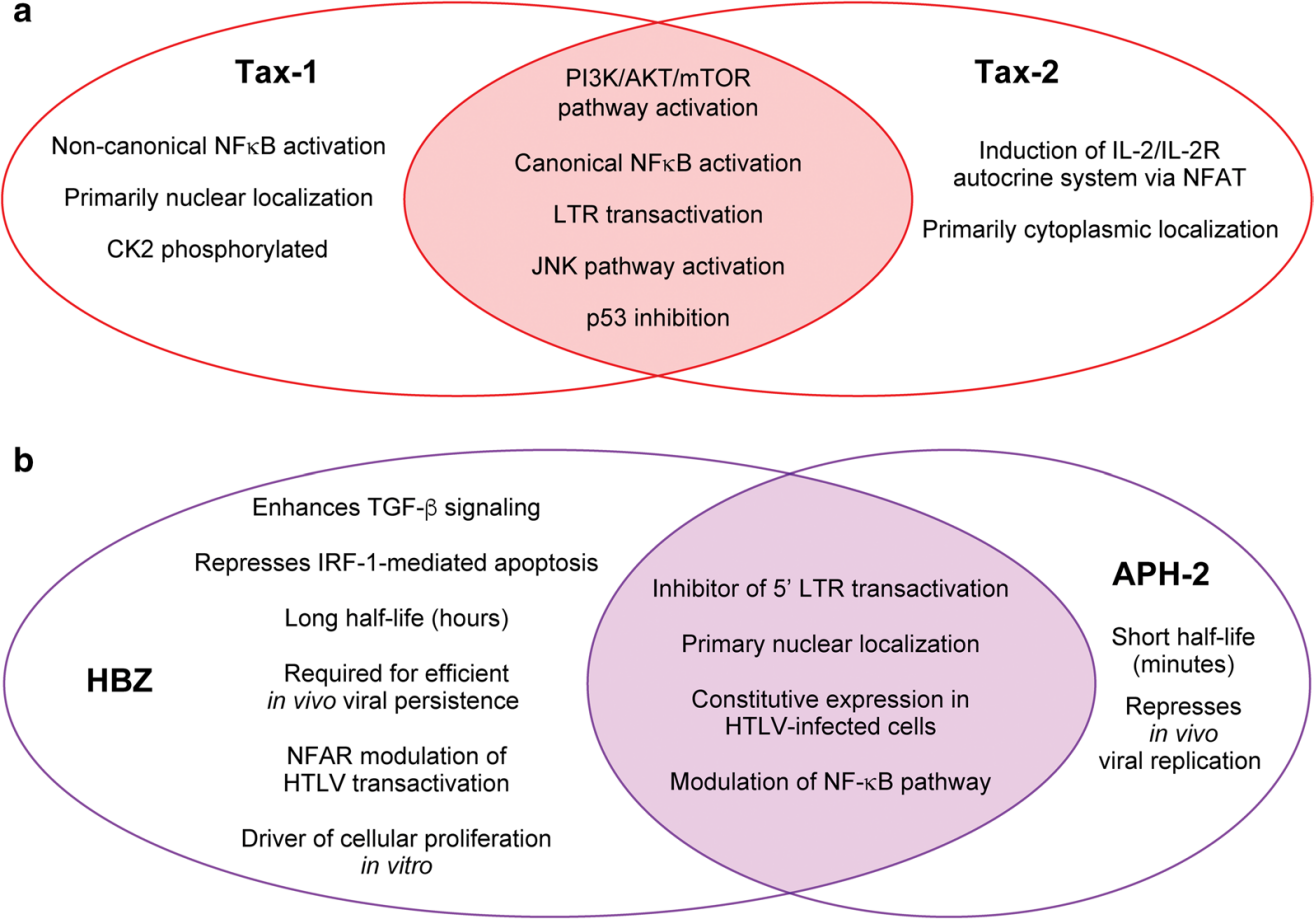
Functional comparison of Tax-1 compared to Tax-2 and HBZ compared to APH-2. **a** Functional comparison of Tax-1 and Tax-2. **b** Functional comparison of HBZ and APH-2

HTLV-1 and HTLV-2 encode the pleiotropic transactivator proteins Tax-1 and Tax-2, respectively, which share 85% amino acid identity [42]. Both proteins contain CREB-activating domains (N-termini), zinc finger domains (N-termini), nuclear localization signals (Tax-1, within first 60 amino acids; Tax-2, within first 42 amino acids), nuclear export signals (amino acids 189–202) and ATF/CREB-activating domains (C-termini regions) (Fig. 2a, b) [42–49]. Unlike Tax-2, Tax-1 has two leucine zipper-like regions (amino acids 116–145 and 225–232) responsible for activation of the canonical and non-canonical NF-κB pathways, a PDZ-binding motif (PBM; C-terminal 4 amino acids), and a secretory signal (C-terminus) [50–52]. Conversely, Tax-2 has a cytoplasmic localization domain (amino acids 89–113), which Tax-1 lacks [53]. Although Tax-1 and Tax-2 have been found in both the nuclear and cytoplasmic compartments of infected cells, the Tax-2 cytoplasmic localization domain explains its primarily cytoplasmic distribution when compared to the primarily nuclear distribution of Tax-1 [47, 49, 53, 54]. Despite their functional domain similarities, the Tax-1 and Tax-2 interactomes and subsequent effects on cellular pathways are divergent (Fig. 3a).

The NF-κB pathway is a major regulator of inflammation, cell survival, and proliferation [55]. Tax-1 interactions with NF-κB were first identified in the late 80s, and since then multiple reviews have outlined the large body of research supporting the role of Tax-1-mediated deregulation of the NF-κB pathway in HTLV-1 cellular transformation/pathogenesis [56, 57]. Tax-1 and Tax-2 differentially interact with the NF-κB pathway, which plays a role in the differential transforming activity in culture and likely contributes to the distinct pathogenesis of HTLV-1 and HTLV-2 [42, 58]. Tax-1 constitutively activates the canonical NF-κB pathway via combinations of interactions with multiple factors, including the IκB kinase complex (IKKα; IKKβ; NEMO/IKKγ), inhibitors of κB (IκBα; IκBβ), RelA, NEMO-Related Protein (NRP), Tax1-binding protein 1 (TAX1BP1), and TAK1-binding-protien 2 (TAB 2) [59–65]. Tax-1 uniquely interacts with the E3 ubiquitin ligase TRAF6, which activates downstream signaling of the NF-κB pathway, whereas Tax-2 does not [66]. Additionally, Tax-1 has been shown to promote the NF-κB pathway via IKK activation through interaction with another E3 ubiquitin ligase, RNF8, and through recruitment of linear ubiquitin chain assembly complex [67, 68]. Tax-2 also activates the canonical NF-κB pathway, albeit through a smaller subset of interacting partners; NEMO/IKKγ, RelA, NRP, and TAB 2 [53, 64, 65, 69]. Tax-1, but not Tax-2, also activates the non-canonical NF-κB pathway by driving NEMO and IKKα-mediated p100 processing to p52 [70, 71]. Additionally, Tax-1, but not Tax-2, was recently shown to induce expression of the immunostimulatory ligand OX40 via interactions with p52/p100 and RelB; components of the non-canonical NF-κB pathway [72]. Both Tax-1 and Tax-2 have been found to associate with plasma membrane-associated lipid raft microdomains, but only Tax-1 has been shown to modulate NF-κB activation via recruitment of IKK subunits through this association [73]. Lastly, Tax-1 and Tax-2 have been shown to interact with IκB kinases, IKKε and TBK1; both of which can play a role in STAT3, NF-κB, and IFNα activation/induction [74, 75].

Tax-1 and Tax-2 activation of the NF-κB pathways, as well as other functions, is also regulated by differential post-translational modification. It has been shown that phosphorylation of Tax-1 is required for nuclear body localization with RelA and activation of the NF-κB and ATF/CREB pathways [76]. Tax-1 is phosphorylated by the serine/threonine kinase CK2 [77].

In addition to phosphorylation, ubiquitylation, SUMOylation, and acetylation have been shown to play roles in Tax-1 localization and function [54, 66, 69, 78–81]. The function of Tax-2 ubuiquitylation and SUMOylation in NF-κB activation has been explored with varied results. A 2012 study by Turci et al. found that ubiquitylation and SUMOylation is of similar importance for both Tax-1- and Tax-2-mediated NF-κB activation [78]. In contrast, a 2013 study by Journo et al. demonstrated that a Tax-2 mutant defective for ubiquitylation and SUMOylation maintained its ability to drive an NF-κB-dependent promoter [66]. Tax-1 acetylation has been shown to promote NF-κB pathway activation with the aforementioned phosphorylation acting as a pre-requisite [81]. Tax-2 has similarly been shown to be acetylated [81].

Tax-1 and Tax-2 drive virus transcription via their respective promoters located in the 5′ LTR. Alteration of ATF/CREB function through the ATF/CREB-activating domains of Tax-1 and Tax-2 is critical for transactivation [82]. Tax-1 has been shown to activate or repress multiple downstream genes through the ATF/CREB pathway [42].

Tax-1 contains a C-terminal PDZ binding motif (PBM) that Tax-2 lacks. It has been shown that this PBM is important for PI3K/AKT/mTOR pathway activation via attenuation of negative regulators PTEN and PHLPP through competitive binding of DLG-1 [83]. The PI3K/AKT/mTOR pathway is a widely studied regulator of cell cycle progression and proliferation. Differential activation of this pathway between Tax-1 and Tax-2 likely contributes to the distinct pathobiology of HTLV-1 and HTLV-2. A recent study demonstrated that the PBM domain for Tax-1 is required to interact with the cellular protein SNX27 [84]. This interaction facilitates the ability of Tax-1 to regulate the localization of the receptor molecule GLUT1 on the surface of cells altering virus production and infectivity.

Tax-1 and Tax-2 have been shown to drive various cellular pathways via activation of MAPKs. Tax has been documented to bind MEKK1, TAK1, and GPS2 which, in turn, play roles in activation of the NF-κB and JNK pathways [85, 86].

Lastly, Tax-1 has been shown to repress the activity of the critical tumor suppressor gene p53 through various pathways including the NF-κB and ATF/CREB pathways discussed above [87]. Tax-2 has also been found to inhibit p53 function [88]. Tax-1 disrupts other cell cycle checkpoint and DNA damage repair systems; these interactions are thoroughly reviewed elsewhere [89]. There is a paucity of comparative information concerning the effects of Tax-2 on these systems.

## HBZ and APH-2

HTLV-1 and HTLV-2, both encode gene products from the antisense strand of the proviral genome, termed HBZ and APH-2, respectively. Like Tax-1 and Tax-2, HBZ and APH-2 feature multiple similarities and differences that likely contribute to the pathogenic potential or lack thereof. The prominent similarities and differences will be discussed in this section and are summarized in Fig. 3b.

The presence of the HTLV-1 antisense transcript HBZ was clearly demonstrated by Gaudray et al. in 2002 after prior identification of a conserved open reading frame in the antisense strand of the HTLV-1 genome [90, 91]. Years later, APH-2 was described as the antisense transcript of HTLV-2 by Halin et al. [92]. Both *HBZ* and *APH*-*2* mRNAs are transcribed from the 3′ LTR and are polyadenylated [92]. HBZ is a nuclear protein with three functional domains: N-terminal transactivation domain, a central modulatory domain, and a C-terminal bZIP domain. APH-2 similarly has a central modulatory domain, but lacks an N-terminal transactivation domain and has a non-conventional C-terminal bZIP domain (HBZ and APH-2 are diagramed in Fig. 2a, b).

HBZ protein represses Tax-mediated proviral transcription through heterodimer formation with CREB, CREB-2, CREM, and ATF-1 [90, 93–95]. This heterodimer formation prevents Tax-1-induced binding of these transcription factors to Tax Responsive Elements (TREs) located in the LTR, blocking sense provirus transcription. APH-2 has been shown to perform a similar function in HTLV-2 through interactions with CREB via its non-conventional bZIP domain [92, 96]. Despite their similar use of ATF/CREB proteins as means to suppress Tax-induced provirus transcription, HBZ possesses far greater inhibitory potential when compared to APH-2. Two potential mechanisms behind this difference in repression ability are the significant difference in protein half-lives (discussed below) and that APH-2 lacks a N-terminal transactivation domain. HBZ has been shown to interact, through its transactivation domain, with the KIX domain of p300/CBP [97, 98]. The binding of HBZ to the KIX domain inhibits Tax-1 interaction with the KIX domain and subsequent CREB-mediated provirus transcription. Another potential mechanism for this difference was described by Murphy et al. in 2016. HBZ and APH-2 were individually found to interact with NFAR; HBZ interaction with NFAR resulted in decreased Tax-mediated transactivation while APH-2 interaction with NFAR did not [99].

Deletion of either antisense protein from their respective HTLV molecular clones has no effect on in vitro immortalization of primary T-lymphocytes. However, antisense protein deletion does result in divergent phenotypes in vivo using a rabbit model of infection. Loss of HBZ lowers the replication and persistence of HTLV-1 infection while loss of APH-2 increased in vivo HTLV-2 replication and proviral load in rabbits [96]. Thus, APH-2 manifests itself as an inhibitor of viral replication, whereas HBZ has evolved additional functions. Both HBZ and APH-2 have been shown to inhibit RelA/p65 activity [100, 101]. HBZ represses IRF-1 transcriptional activity while APH2 appears to promote IRF-1 [100]. Thus, HTLV-2 may be more susceptible to IRF-1-mediated apoptosis [100]. HBZ, but not APH-2, enhances TGF-β signaling and APH-2 has a considerably shorter half-life than that of HBZ (approximately 20–30 min vs 2–6 h) [100]. The drastic difference in HBZ and APH-2 half-life may be the result of differential mechanisms of stability maintenance. It has been shown that HBZ stability is largely regulated by UBR5, an E3 ubiquitin ligase, whereas the stability of APH-2 has been shown to be controlled by PML nuclear bodies in a sumoylation-dependent manner [102].

Several other functions have been attributed to HBZ. These include induction of genomic instability through double strand breaks [95], enhancement of hTERT expression through JunD [95], suppression of apoptotic factor Bim [103], activation of the mTOR pathway [104], inactivation of tumor suppressor p53 [95], and upregulation of non-canonical Wnt signaling, and suppression of canonical Wnt signaling [105]. These, and other alterations to cellular metabolism, likely all contribute to HBZ-induced HTLV-1 pathobiology, but direct comparisons concerning these functions between HBZ and APH-2 have yet to be explored.

## Other genes

HTLV-1 and HTLV-2 express other regulatory and accessory genes including *Rex*-*1/Rex*-*2*, *p21Rex/truncated Rex*, *p30/p28*, and *p12/p10*. HTLV-1 also expresses p13 and p8 while HTLV-2 expresses p11, these three proteins do not have a homologue in the opposing virus. This section will briefly touch on the remaining gene products of HTLV.

### Rex-1 and Rex-2

The main function of Rex in the viral lifecycle is to promote the export of the full length unspliced mRNA from the nucleus [106]. This is required to bypass the cellular mechanisms that retain intron containing mRNAs in the nucleus. Rex promotes viral mRNA export by binding to a mRNA stem loop structure known as the Rex responsive element (RxRE) that is present in the LTR region of both HTLV-1 and HTLV-2 [107]. Upon Rex binding to the RxRE, multimerization of the Rex protein will occur, which promotes an interaction with CRM-1 [108]. Together with CRM-1, Rex then completes the nuclear export of viral mRNAs, after which Rex will shuttle back to the nucleus.

### Rex isoforms

HTLV-1 expresses one isoform of Rex known as p21Rex, while HTLV-2 expresses several isoforms known as truncated Rex (tRex). HTLV-2 tRex is expressed from two different mRNAs and via different initiation codons resulting in four distinct isoforms between the sizes of 17 and 22 kDa [109]. Both p21Rex and tRex lack the N-terminal domains of the Rex proteins required for binding to the RxRE, therefore these proteins are not capable of interacting with viral mRNAs. It was predicted that both p21Rex and tRex could inhibit the function of the full-length Rex proteins [110]. While this has been confirmed for tRex, no evidence exists for this function of p21Rex [110].

#### p30 and p28

p30 and p28, expressed by HTLV-1 and HTLV-2 respectively, each function to retain the spliced *Tax/Rex* mRNA in the nucleus resulting in inhibition of virus production [111, 112]. p28 has been shown to be required for in vivo viral persistence in the rabbit model of infection [113]. Interestingly, p30 is dispensable for viral persistence in the rabbit model of infection while it is required for infectivity in macaques [114]. p30 inhibits Tax-1-mediated transcription via a competitive binding event with CBP/p300, however, p28 does not appear to have this capability [115]. HTLV-1 p30 has several other reported functions that have not been documented for p28. These functions include modulating DNA damage recognition and down-regulation of toll-like receptor 4 [116, 117]. Few studies have been performed on p28, leaving it as a potentially valuable target for information regarding the differing pathologies of HTLV-1 and HTLV-2.

#### p12/p8 and p10

The last gene products with similar sequence and function between the two viruses are HTLV-1 p12 and HTLV-2 p10. p12 is a membrane bound protein that is localized to the endoplasmic reticulum (ER) and Golgi. p12 appears to play a role in dendritic cell infection, but deletion of p12 from the provirus does not alter PBMC immortalization in vitro or viral persistence in vivo [114]. p12 reduces expression of ICAM-1 and ICAM-2 on the surface of infected cells, which prevents NK cell-mediated death [118]. p12 can be proteolytically cleaved into a carboxyl terminal product, p8, which localizes at the cell membrane due to the removal of the ER retention signal [119]. p8 has been shown to mediate HTLV-1 transmission via activation of the lymphocyte function-associated antigen-1, which promotes cell-to-cell contact of T-cells and increases the potential for viral transmission [119]. HTLV-2 p10 has been shown to bind to MHC-1 but to date no other functions have been identified [120]. HTLV-2 also does not express a homologue to HTLV-1 p8.

#### p13

HTLV-1 p13 is a mitochondrial-associated protein of 87 amino acids, which is identical to the carboxyl-terminal 87 amino acids of p30 [121]. Mutations of p13 in HTLV-1 virions do not alter viral infectivity in inoculated rabbits [122]. p13 expression has been tied to increased reactive oxygen species production and apoptosis [123]. HTLV-2 does not express a homologue to p13.

#### p11

p11 is a unique protein expressed by HTLV-2. p11 is expressed from the same mRNA transcript as p10. p11 has been shown to bind to MHC-1 potentially modulating the immune response [120].

## Tropism and clonality

The in vivo tropism of HTLV-1 and HTLV-2 differ, with HTLV-1 being primarily detected in CD4^+^ T-lymphocytes, and HTLV-2 in CD8^+^ T-lymphocytes [124, 125]. Previous studies have further investigated this divergent tropism and both viral and cellular determinants have been suggested as potential contributors [126, 127]. GLUT1 and NRP1 serve as receptor molecules for both HTLV-1 and HTLV-2, while HTLV-1 uses HSPG as an additional co-receptor [27]. CD4^+^ T-cells demonstrate high levels of HSPG expression and minimal GLUT1, whereas CD8^+^ T-cells demonstrate the opposite. A recent in vivo study found that the tropism for CD4^+^ and CD8^+^ T-lymphocytes was equivocal 1-week post-inoculation of New Zealand White rabbits with HTLV-1 and HTLV-2 [127]. A similar result was demonstrated via in vitro PBMC immortalization assays early after co-culture [127]. In cell culture, the CD4^+^:HTLV-1 and CD8^+^:HTLV-2 tropisms were established several weeks after infection [127]. This result clearly suggests a post entry event driving the preferential expansion.

**Fig. 4:**
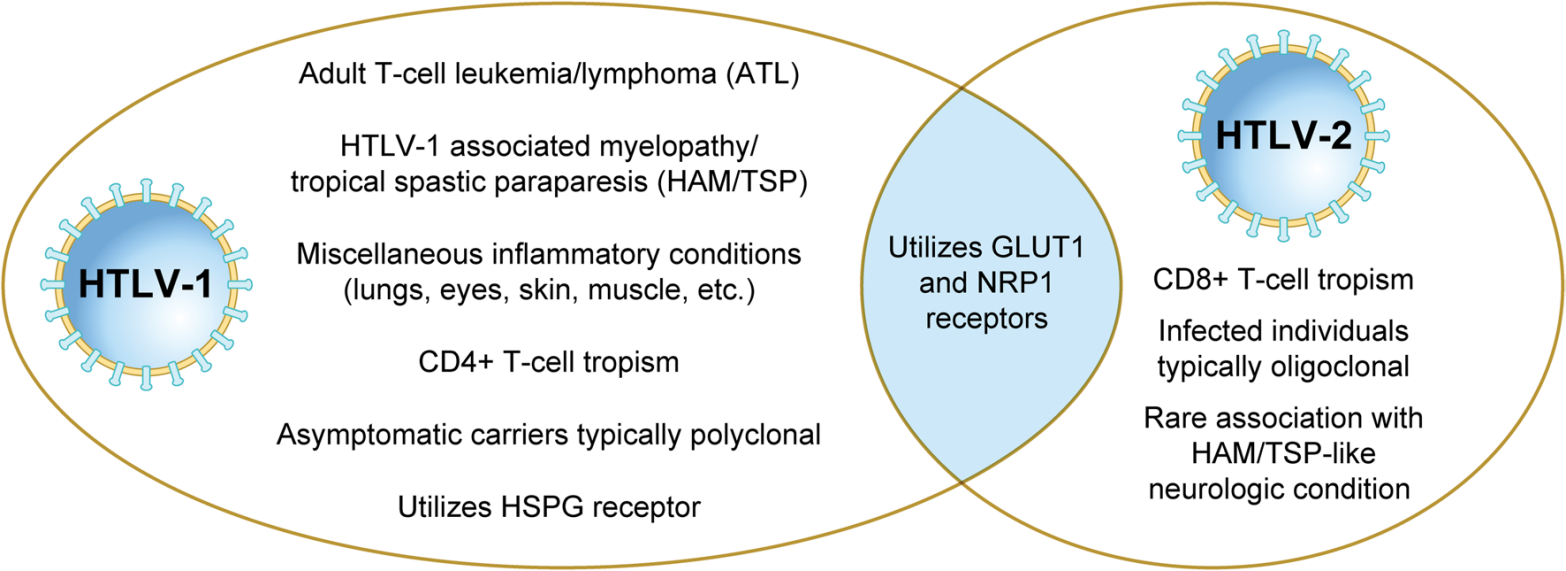
Comparison of HTLV-1 and HTLV-2 pathobiology

Infection with either HTLV-1 or HTLV-2 have been shown to result in clonal proliferation of T-cells [128, 129]. An HTLV-1 infected host has an estimated 28,000 clones circulating [130]. In ATL, 91% of the dominant clones contain a single provirus with integration site characteristics that resemble those of low-abundance clones found in both ATL cases and asymptomatic individuals [131]. This suggests that oligoclonal expansion does not necessarily result in malignant transformation. HTLV-2 infected individuals typically carry a small number of markedly expanded clones supporting the notion that oligoclonality does not necessarily translate to malignant potential [132]. Differences in HTLV-1 and HTLV-2 tropism, clonality, and pathobiology are summarized in Fig. 4.

## Conclusion

HTLV-1 and HTLV-2 are highly related viruses with different pathobiology. While being closely related with sequence and genomic structure, there exists several differences between HTLV-1 and HTLV-2. Several studies within the field have suggested these differences may help explain the different disease outcome between these two viruses. It is worth noting that the smaller populations of individuals infected with HTLV-2 may mask a potential disease caused by this virus, but in vivo and in vitro work by members of the HTLV research community clearly demonstrate the lower transforming capacity of HTLV-2. The regulatory Tax proteins and antisense-derived proteins of the two viruses are the most well compared of HTLV-1 and HTLV-2.

The few differences that exist between HTLV-1 and HTLV-2 require further intense study. The complete exploration of what makes HTLV-1 pathogenic compared to HTLV-2 would open many avenues to fight this pathogenic capability and improve the livelihoods of individuals infected with HTLV-1. Also, increased understanding of how HTLV-1 causes cancer has broad impact on the cancer field as a whole, potentially uncovering new therapies for other cancer types.

## Data Availability

Not applicable.

## Abbreviations

APH-2: antisense protein of HTLV-2
ATL: adult T-cell leukemia
Env: envelope
Gag: group-specific antigen
GLUT1: glucose transporter type 1
HAM/TSP: HTLV-1-associated myelopathy/tropical spastic paraparesis
HBZ: HTLV-1 bZIP transcription factor
HSPG: heparan sulfate proteoglycan
HTLV: human T-cell leukemia virus
LTR: long terminal repeat
NRP1: neuropilin 1
PBM: PDZ-binding motif
Pol: polymerase
Pro: protease
Rex: rex
RxRE: rex responsive element
SU: surface
Tax: transactivator from the X-gene region
TAX1BP1: Tax1-binding protein 1
TM: transmembrane
TRE: tax responsive element
tRex: truncated Rex
NRP: NEMO-Related Protein
TAB 2: TAK1-binding-protien 2
